# Role of survey response rates on valid inference: an application to HIV prevalence estimates

**DOI:** 10.1186/s12982-018-0074-x

**Published:** 2018-03-05

**Authors:** Miguel Marino, Marcello Pagano

**Affiliations:** 1 Department of Family Medicine, Oregon Health and Science University, 3181 SW Sam Jackson Park Road, Mailcode: FM, Portland, OR 97239 USA; 2 Division of Biostatistics, School of Public Health, Oregon Health and Science University – Portland State University, Portland, OR USA; 3 Department of Biostatistics, Harvard T.H. Chan School of Public Health, Boston, MA USA

**Keywords:** HIV reporting, HIV testing, Missing at random, Nonresponse, Survey bias

## Abstract

**Background:**

Nationally-representative surveys suggest that females have a higher prevalence of HIV than males in most African countries. Unfortunately, these results are made on the basis of surveys with non-ignorable missing data. This study evaluates the impact that differential survey nonresponse rates between males and females can have on the point estimate of the HIV prevalence ratio of these two classifiers.

**Methods:**

We study 29 Demographic and Health Surveys (DHS) from 2001 to 2010. Instead of employing often used multiple imputation models with a Missing at Random assumption that may not hold in this setting, we assess the effect of ignoring the information contained in the missing HIV information for males and females through three proposed statistical measures. These measures can be used in settings where the interest is comparing the prevalence of a disease between two groups. The proposed measures do not utilize parametric models and can be implemented by researchers of any level. They are: (1) an upper bound on the potential bias of the usual practise of using reported HIV prevalence estimates that ignore subjects who have missing HIV outcomes. (2) Plausible range intervals to account for nonresponses, without any additional parametric modeling assumptions. (3) Prevalence ratio inflation factors to correct the point estimate of the HIV prevalence ratio, if estimates of nonresponders’ HIV prevalences were known.

**Results:**

In 86% of countries, males have higher upper bounds of HIV prevalence than females, this is consonant with males possibly having higher infection rates than females. Additionally, 74% of surveys have a *plausible* range that crosses 1.0, suggesting a plausible equivalence between male and female HIV prevalences.

**Conclusions:**

It is quite reasonable to conclude that there is so much DHS nonresponse in evaluating the HIV status question, that existing data is plausibly generated by the situation where the virus is equally distributed between the sexes.

## Background

The use of large-scale surveys to estimate local and national prevalence of disease, or other population characteristics, typically encounter nonresponse on the status of the disease [19]. If the aim of a study is to make comparisons of disease prevalence between two groups, nonresponse in survey items may introduce bias in the comparison, especially if the non-response rate differs in the two groups. A variety of methods are available to address nonresponse including weighting adjustments to account for total nonresponse and imputation methods to assign values to missing response items [4]. The development of these suite of methods are important because accurate national prevalence estimates are needed for monitoring the pandemic, policy formulation, planning and evaluating treatment interventions.

Consider the motivating example of estimating HIV prevalence in African countries. Early published estimates of HIV prevalence for African countries were derived from sentinel surveillance which have shown to over—and under—estimate the prevalences when measured this way [2, 13, 14, 18]. This limitation has led to the estimation of HIV prevalence through, presumably more accurate, national population-based surveys [27].

The largest national population-based surveys designed to estimate HIV prevalence in the developing world are the Demographic and Health Surveys (DHS). The DHS aim to be a nationally representative, population-based set of surveys including HIV prevalence data for multiple countries of Africa, in part because of its use of blood collected for HIV testing [5, 9]. One of the claimed major advantages of a DHS is that it provides researchers the ability to estimate HIV prevalence for the general population and for certain subgroups, such as sex and age groups.

Throughout Africa, empirical evidence suggests that despite large HIV prevalence differences between countries, females consistently have higher HIV prevalence than males [1, 11, 15, 21, 29, 30, 40]. Theories have been advanced to explain that the observed sex difference of HIV prevalence in Africa is driven by multiple factors [25]. Biological differences between males and females are thought to explain the sex difference in HIV prevalence [10, 31, 32]. It has been observed that younger females tend to have older male sexual partners who are at higher risk of HIV [16, 17]. Biologically, it is also believed that there is higher efficiency of transmission from males to females than vice versa [28]. It has also been reported that socially, females in African countries have less authority in controlling the dynamics of sexual behavior [3, 36]. That such a sex differential exists is not surprising given the sex differences in the perception of health services that have existed [35, 39].

The aim of our study is to evaluate the role of yet another factor in the posited observed differences, namely nonresponse (refusal to consent to being tested), as a potential explanation that could modify the observed sex differences in HIV prevalence estimates in 29 DHSs from 2001 to 2010. It is common for population-based surveys to experience non-ignorable nonresponse on HIV relevant variables due to refusal to provide a blood sample for HIV testing, subject absenteeism, subject mobility and general non-consent. Depending on actual reasons why subjects fail to provide HIV testing information, survey nonresponse in the numbers experienced in the DHS, cannot be ignored because of the potential to bias estimates of HIV prevalence. We hypothesize that the differential nonresponse rates between males and females can play a sizable role in the supposed differences of HIV prevalence; certainly when the basis for such claimed sex differences are the DHS. Several methods to account for nonresponse have been proposed including weighting adjustments [26], Heckman-type selection models [6], mathematical modeling [33] and multiple imputation [27]. The most common approach to address this differential nonresponse issue is to perform multiple imputations on the missing data [11, 29, 30, 42]. These studies conclude that missing subjects typically have higher HIV prevalence but the overall effect of nonresponse is negligible and the observed female to male HIV prevalence ratio changes minimally. The biggest limitation of using multiple imputation in this setting is that these studies make a ‘Missing at Random’ (MAR) assumption [23] that implies that the HIV status of nonresponders is the same as responders with the same observed covariates. However, if an unobserved covariate is correlated with the decision to get tested and HIV status, this condition is violated and multiple imputation would not be a suitable method to address this nonresponse issue. In this HIV example, this is likely to be the case as individuals who suspect or know that they are HIV positive may not adhere to being tested. HIV remains a highly stigmatizing disease in many African countries and subjects may decide not to participate in the survey because of a fear of discovering their status, or having their status possibly revealed and not seeing any advantage in participating in the survey [41]. This issue is compounded when the prevalence of HIV testing is substantially different between males and females [34].

As a companion to multiple imputation when it is not appropriate to assume response is MAR, we discuss three statistics that are straightforward and intuitive to perform to study the sensitivity of inference when there is no single accepted class of assumptions about the nonresponse mechanism. First, we present an upper bound on the potential bias of sex-specific HIV prevalence estimates when using only the response data and show that this upper bound depends on the amount of nonresponse in males and females. Second, we introduce the concept of plausible range to this argument, which studies the effect of nonresponse on the estimate of the sex HIV prevalence ratio without any additional modeling assumptions. Finally, we derive an HIV prevalence ratio inflation factor that would correct the estimate of the HIV prevalence ratio if the nonresponders HIV prevalence were known.

## Methods

### Study population and data

The standard DHSs include information about house member demographic characteristics including age and sex. Since 2001, a subset of DHSs have included HIV testing results to produce supposed nationally representative estimates of HIV prevalence. We study the (first) 29 DHS available to us as of writing this paper that performed HIV-related measurements.

We evaluate the sex-specific characteristics of the national surveys including the number eligible for HIV testing, the HIV testing response rate, the age range, the HIV prevalence estimate and the ratio of female to male HIV prevalence. HIV prevalence is defined as the number of subjects with a positive test result for HIV-1 or HIV-2 over the number tested for HIV. HIV response rate was taken to be the number of subjects with an HIV test result over the number eligible for HIV testing. We define nonresponse as being eligible for HIV testing and having a missing observation on HIV testing which could have been due to refusal to be tested, not being available during the interview, or any other factor. Our analyses apply individual HIV sampling weights that account for the DHS sample design [38].

### Statistical analysis

To evaluate the impact of missing HIV outcomes on the HIV prevalence estimates we present three informative quantities, none of which requires any further modeling assumptions to be valid. The first measure we present is the upper bound on nonresponse bias. Following a similar framework to that proposed by Cochran [7], let $p$ denote the true HIV prevalence for a country. Denote by $w$ the proportion of nonresponders in a survey. Associated with the nonresponders is their HIV prevalence that we label $p_{nr}$. The HIV prevalence of the population, $p$, can be expressed by the following composition formula:

1 $$ {\text{p}} = {\text{p}}_{\text{nr}} {\text{w}} + {\text{p}}_{\text{r}} (1 - {\text{w}}) $$

where $p_{r}$ is the prevalence of HIV for the subjects who consented to HIV testing (i.e. responders).

Equation (1) identifies how the proportion of the nonresponders in the population plays a role in the estimation of national HIV prevalence. Using this formula, we can calculate the bias induced by using the HIV prevalence of the fully observed subjects as the true HIV prevalence. We have that the bias,

$$ {\text{Bias}} = {\text{w}}\left| {{\text{p}}_{\text{nr}} - {\text{p}}_{\text{r}} } \right|. $$

depends on the amount of nonresponse ($w$) and the difference in HIV prevalence between the population that responds and those who do not respond to the survey. Furthermore, because the term $\left| {p_{nr} - p_{r} } \right|$ is between zero and one, $w$ provides an upper bound on the bias,

2 $$ {\text{Bias}} \le {\text{w}} $$

that makes it evident that the difference between the true HIV prevalence and the prevalence of the fully-observed subjects will be at most the proportion of nonresponses in the population. Given that $p_{r}$ is known, a sharper bound for the bias is simply

3 $$ {\text{Bias}} \le {\text{w}}\left( {1 - {\text{p}}_{\text{r}} } \right) $$

when it is assumed that the prevalence of nonresponders is larger than that of responders (i.e. $p_{nr} > p_{r}$).

The second quantity we present is the plausible range. Instead of addressing the nonresponse issue through scientifically questionable MAR imputation models, we propose to focus on the effect this issue has by implementing the metric of plausible range to more honestly evaluate the information in the survey. Inspired by the work of Cochran et al. [8] we first look at the estimated prevalence if we assume all missings were to test negative. Then the estimated prevalence if all missings were to test positive. We construct the HIV prevalence ratio plausible range comparing females to males as:

4 $$ PR = (PR^{ - } ,PR^{ + } ) $$

where $PR^{ - }$ denotes the estimated HIV prevalence ratio when all the missing HIV responses for males and females are assigned a negative test result and $PR^{ + }$ denotes the estimated HIV prevalence ratio when all nonresponders are assigned a positive HIV test result. Formulations for $PR^{ - }$ and $PR^{ + }$ can be found in the Appendix. The plausible range interval is a measure of how missing HIV outcomes potentially affect the point estimate of the sex HIV prevalence ratio. A narrow plausible range suggests that the effect of nonresponse on the point estimate of the prevalence ratio is minimal. Additionally, the location of the plausible range interval is important. If the plausible range interval crosses the null value of 1.0, it is plausible that the HIV prevalence for females is equivalent to the HIV prevalence for males *even before taking into account the standard error of the prevalence ratio*. We present this statistic as a conservative guide and not as a worst-case scenario. Of course the worst-case scenario would have all male missings be in the one direction and all the female missings be in the other direction, but we do not consider this possibility, preferring to believe that the reasons for missingness are more likely to be similar between the sexes than completely opposite.

The next measure, the prevalence ratio inflation factor, allows us to quantitate differential sex-behavior. We explore the joint role that nonresponse rates and nonresponders HIV prevalence plays on the estimate of the sex prevalence ratio. After some algebra (shown in the Appendix), the true HIV prevalence ratio between females and males ($RR_{adj}$) adjusting for the HIV characteristics of nonresponders can be expressed as

5 $$ RR_{adj} = RR_{obs} \times \left[ {\frac{{1 + w^{F} (R^{F} - 1)}}{{1 + w^{M} (R^{M} - 1)}}} \right] $$

where $RR_{obs}$ is the observed HIV prevalence ratio between females and males, $w^{F}$ is the proportion of female nonresponders, $R^{F}$ is the ratio of HIV prevalences of nonresponders to responders for the female population, $w^{M}$ is the proportion of male nonresponders, and $R^{M}$ is the ratio of HIV prevalences of nonresponders to responders for the male population. The bracketed term on the right side Eq. (5) is what we term the prevalence ratio inflation factor, which depends on male and female nonresponse rates (available from the survey) and the HIV prevalence ratio between nonresponders and responders (which is unavailable from the survey because the HIV prevalence of nonresponders is unknown). If a reliable estimate of the HIV prevalence for female and male nonresponders could be obtained, then it would be possible to adjust the observed prevalence ratio to obtain a more representative female to male HIV prevalence ratio that accounts for missing HIV outcomes using Eq. (5).

## Results

Sex-specific observed HIV prevalence estimates and nonresponse rates for each of the 29 DHS are presented in Table 1. We see a clear pattern of higher reported HIV prevalence among females when compared to males. Of the 29 DHS analyzed, 26 had an HIV female:male prevalence ratio greater than one. The three highest reported HIV prevalence ratios among the surveys were in Cote d’Ivoire, Senegal and Ethiopia. In these three countries the HIV response rates for males (that is, males who consented to being tested) were considerably lower than most DHS. Across all the DHS analyzed, males had a higher HIV nonresponse rate compared to females except for the Congo Brazzaville survey. The average HIV testing nonresponse rate across all surveys for females was 13.4% (range 2.7–29.6%; median: 12.3%) and for males 20.2% (range: 4.4–36.7%; median: 20.1%).

**Table 1:** HIV testing response percentages and observed HIV prevalence estimates for 29 DHS with testing by sex

Country	Year	Females				Males				HIV F:M Prev ratio	*p* value
		Age range	# eligible HIV testing	HIV Resp %	Obs HIV Prev %	Age range	# eligible HIV testing	HIV Resp %	Obs HIV Prev		
Burkina Faso	2003	15–49	4575	92.3	1.83	15–59	3984	85.8	1.94	0.94	0.77
Cameroon	2004	15–49	5703	92.1	6.75	15–59	5676	89.9	3.91	1.73	< 0.01
Congo Brazzavile	2009	15–49	6804	93.3	4.12	15–49	6143	93.7	2.06	2.00	< 0.01
Congo DR	2007	15–49	5127	91.0	1.62	15–59	4985	88.4	0.92	1.76	0.02
Cote d’Ivoire	2005	15–49	5772	78.6	6.41	15–49	5148	75.6	2.86	2.24	< 0.01
Ethiopia	2005	15–49	7142	83.4	1.86	15–59	6778	75.5	0.91	2.04	< 0.01
Ghana	2003	15–49	5949	89.1	2.70	15–59	5345	79.9	1.66	1.62	< 0.01
Guinea	2005	15–49	4189	92.4	1.87	15–59	3360	88.0	1.09	1.72	0.02
Kenya	2003	15–49	4303	76.3	8.70	15–54	4183	70.3	4.71	1.85	< 0.01
Kenya	2008/09	15–49	4418	86.4	7.98	15–54	3910	79.9	4.55	1.75	< 0.01
Lesotho	2004	15–49	3758	80.7	26.37	15–59	3305	68.0	18.94	1.39	< 0.01
Lesotho	2009	15–49	4112	93.8	26.68	15–59	3494	88.2	18.44	1.45	< 0.01
Liberia	2007	15–49	7448	87.7	1.92	15–49	6476	80.9	1.22	1.57	0.01
Malawi	2004	15–49	4071	70.4	13.32	15–54	3797	63.3	10.23	1.30	< 0.01
Malawi	2010	15–49	8174	90.8	12.88	15–54	7783	84.1	8.39	1.54	< 0.01
Mali	2001	15–49	4556	84.8	2.05	15–59	4062	75.6	1.33	1.54	0.07
Mali	2006	15–49	5157	93.2	1.53	15–59	4643	85.0	1.14	1.34	0.20
Mozambique	2009	15–64	6749	87.7	12.67	15–64	5319	83.0	9.04	1.40	< 0.01
Niger	2006	15–49	4899	92.0	0.70	15–59	3839	85.2	0.72	0.97	0.91
Rwanda	2005	15–49	5837	97.3	3.61	15–59	4959	95.6	2.24	1.61	< 0.01
SaoTome/Principe	2008/09	15–49	2913	89.7	1.29	15–59	3047	72.5	1.79	0.72	0.26
Senegal	2005	15–49	5350	84.5	0.89	15–59	4375	75.5	0.43	2.05	0.05
Sierra Leone	2008	15–49	3954	89.5	1.73	15–59	3541	86.7	1.16	1.49	0.10
Swaziland	2006/07	15–49	5301	87.2	31.12	15–49	4675	77.6	19.67	1.58	< 0.01
Tanzania	2003/04	15–49	7154	83.4	7.70	15–49	6196	77.1	6.26	1.23	0.01
Tanzania	2007/08	15–49	9735	89.5	6.61	15–49	7935	79.8	4.56	1.45	< 0.01
Zambia	2001/02	15–49	2689	79.3	17.79	15–59	2418	73.3	12.62	1.41	< 0.01
Zambia	2007	15–49	7408	77.1	16.09	15–59	7146	72.3	12.29	1.31	< 0.01
Zimbabwe	2005/06	15–49	9870	75.9	21.12	15–54	8761	63.4	14.75	1.43	< 0.01

*Resp* response, *Obs* observed, *Prev* prevalence, *F:M* female to male; *p* value testing equivalence of observed prevalence between female and male subjects

### Upper bound on nonresponse bias

**Fig. 1:**
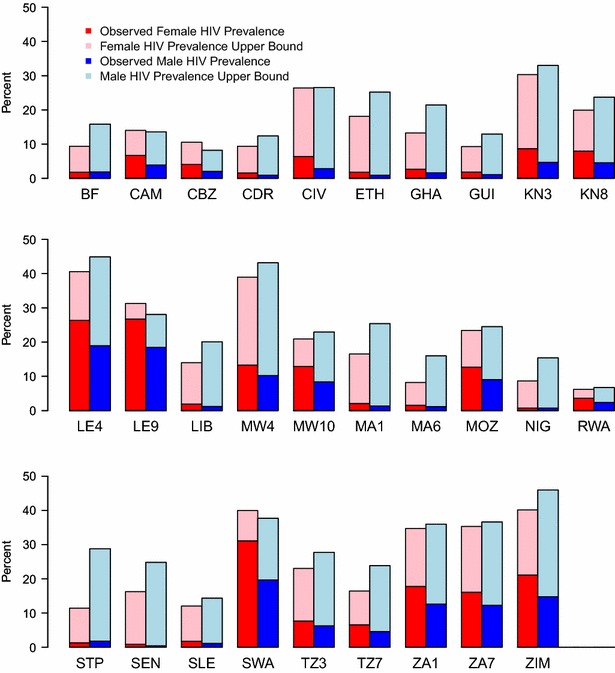
Bar plots of female and male observed HIV prevalence and the upper bound of the HIV prevalence for each of the 29 DHS. Dark red denotes observed female HIV prevalence while light red denotes the female HIV prevalence upper bound. Dark blue denotes observed male HIV prevalence while light blue denotes the male HIV prevalence upper bound. *Note* Letters define the country and if the country had more than one DHS, the last digit of the survey year is added at the end of the country letters. The upper bound used in these estimations are derived from Eq. (3)

In order to avoid having the older male age groups influence the results, we restrict the sample to subjects in the 15–49 age range. We show bar plots in Fig. 1 of the reported HIV prevalence for males and females and their HIV prevalence upper bound using Eq. (3) for 29 DHS country surveys. From Fig. 1 we observe that the upper bound on the HIV prevalence estimate is dependent on the nonresponse rate. As expected, countries with high nonresponse rates have a larger upper bound and, importantly, this size varies between males and females. The bar plots from Fig. 1 identify 25 out of 29 countries that have a higher HIV prevalence upper bound for males than females, suggesting that it is possible that the HIV prevalence ratio can be less than one, reversing the direction of the observed sex gap in HIV prevalence.

Countries with low observed HIV prevalence estimates typically have higher male HIV upper bounds than females. In some instances, the upper bounds on HIV prevalence are twice the size for males compared to females. For example, Senegal has an observed HIV prevalence ratio of 2.07 suggesting that females are twice as likely to test HIV positive compared to males. If the upper bounds are achieved for males and females in the Senegal survey, this would result in an HIV prevalence ratio of 0.66, making females 33% less likely to test positive for HIV than males. Of course, a whole range of ratio values between those two extremes is plausible.

**Fig. 2:**
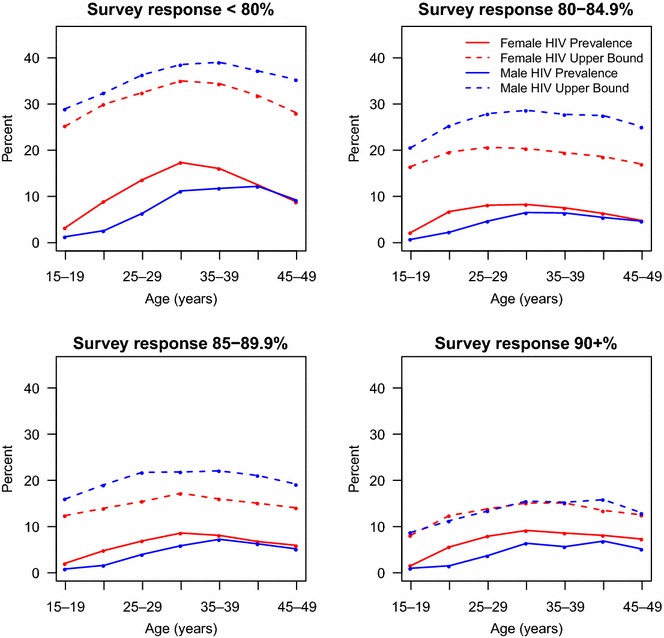
Line plots of HIV prevalence and the upper bound of HIV prevalence averaged across countries with survey response < 80, 80–84.9, 85–89.9 and 90+%. Solid red lines are female HIV prevalence estimates, dashed red lines are the female HIV prevalence upper bound. Solid blue lines are male HIV prevalence estimates, dashed blue lines are the male HIV prevalence upper bound. *Note* The upper bound used in these estimations are derived from Eq. (3)

We also explore how the HIV prevalence and its upper bound vary for males and females across different age subgroups. For the 29 DHSs studied, we group country surveys into four categories, depending on their HIV testing response rates. Within each category, we take the weighted average of HIV prevalence and upper bound by age group. We plot the weighted average of observed HIV prevalence and upper bound across age groups in Fig. 2. We see that for surveys with low response rate, the observed difference between female and male HIV prevalence is large. For surveys with higher response rate (> 85%, for example), the observed HIV prevalence difference is smaller across age groups. Additionally, the upper bound of HIV prevalence is consistently higher across all age groups in each of the four survey response categories. A telling finding is that as HIV testing response rates increase, the upper bounds for males and females HIV prevalences converge (i.e. are equivalent across all age groups).

### Plausible range

**Fig. 3:**
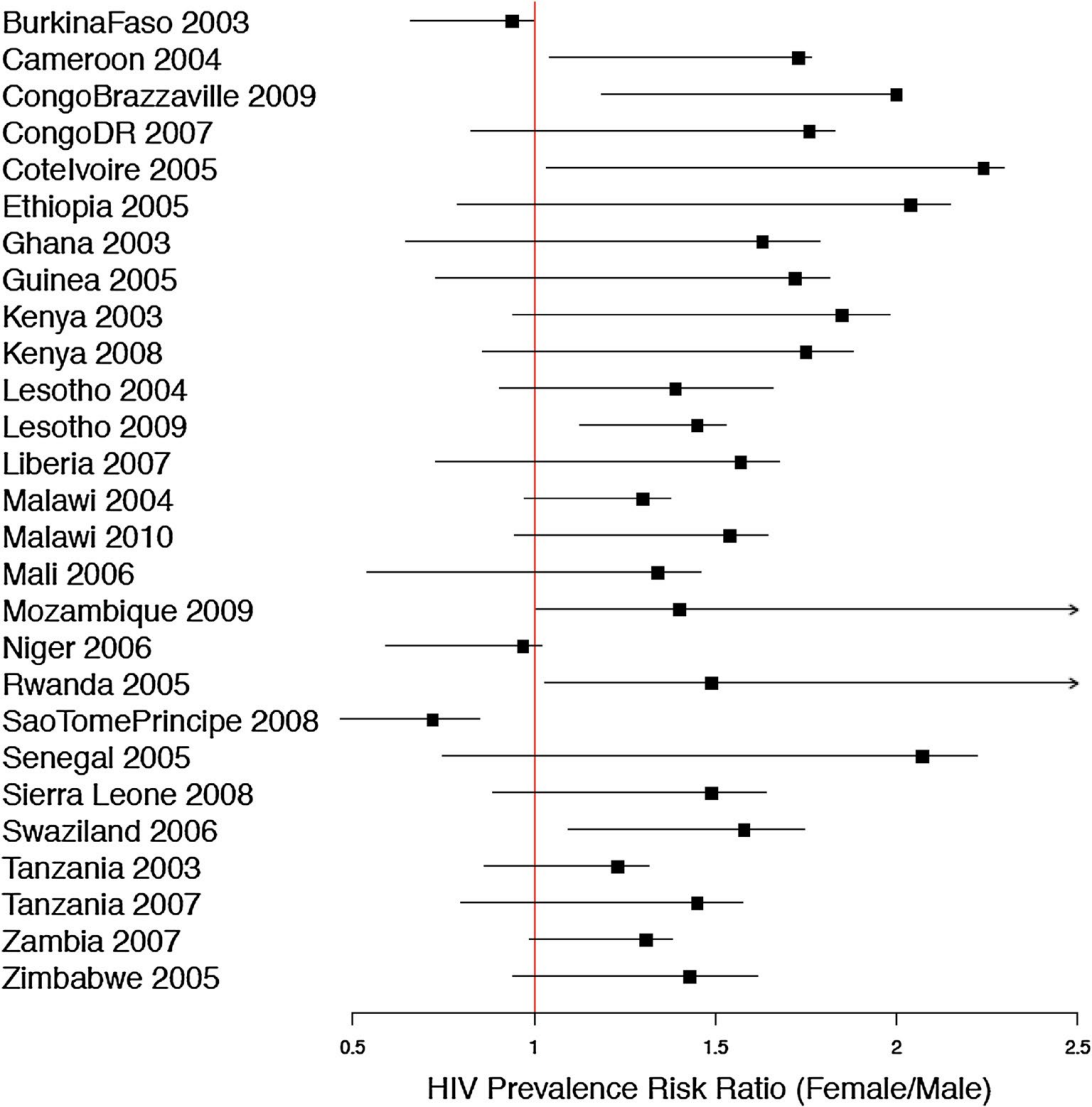
Plausible range plot for the HIV prevalence female to male prevalence ratio for 27 DHS. *Note* The left endpoint of the interval is the plausible value of the prevalence ratio if all nonresponders tested positive. The right endpoint of the interval is the plausible prevalence ratio value if all nonresponders tested negative. The solid square symbol is the observed prevalence ratio for the particular survey. These intervals only display some of the consequences of the missing data. They do not display the sampling uncertainty

Figure 3 plots the plausible range for 27 DHS (two surveys were excluded because individual sampling weights could not be reliably used for nonresponders). From Fig. 3, we note that 20 of the 27 surveys (74%) had a plausible range that crossed the value of 1.0. With the exception of Sao Tome and Principe, the plausible range intervals that did not cross the null value had intervals that were above 1.0 (Cameroon, Congo Brazzaville, Cote d’Ivoire, Lesotho 2009, Rwanda and Swaziland). We also note that for every DHS except the Mozambique and Rwanda surveys, the point estimate of the HIV prevalence ratio is skewed to the right of the plausible range interval. This suggests that the prevalence ratio is more sensitive to nonresponder’s positive HIV test results. The point estimate of HIV prevalence ratio is likely to tend to the null if we believe that the nonresponders are mostly HIV positive individuals.

### Prevalence ratio inflation factor

An important factor in the estimation of the HIV prevalence ratio is the HIV prevalence of female and male nonresponders. Equation (5) can be used to obtain an estimate of the true HIV prevalence ratio that accounts for sex-specific nonresponse rates and nonresponders HIV prevalence. For example, the reported HIV prevalence in Zimbabwe for females was 21.1% and for males 14.6%. The reported HIV prevalence ratio is thus 1.45 suggesting that females are 45% more likely to have HIV than males in 2005–2006. The response rate for females (75.9%) was higher than males (63.6%). If the HIV prevalence for the 24.1% of females who did not respond and the 36.4% of males who did not respond could be estimated, then we could use Eq. (5) to obtain an adjusted HIV prevalence ratio. For the sake of illustration, suppose that the HIV prevalence of nonresponders could be estimated and is 25.0% for both males and females. Using this information and Eq. (5), we obtain an adjusted HIV prevalence ratio:

$$ \begin{aligned} RR_{adj} & = RR_{obs} \times \left[ {\frac{{1 + w^{F} (R^{F} - 1)}}{{1 + w^{M} (R^{M} - 1)}}} \right] \\ RR_{adj} & = 1.45 \times \left[ {\frac{{1 + 0.241{\kern 1pt} (1.18 - 1)}}{1 + 0.364(1.72 - 1)}} \right] \\ RR_{adj} & = 1.20 \\ \end{aligned} $$

The Zimbabwe HIV prevalence ratio changes from 1.45 to 1.20. This exercise illustrates the importance of obtaining reliable estimates of the subjects who do not consent to HIV testing. Unfortunately, we do not know the true HIV prevalence of the nonresponders, but we can use Eq. (5) to assess how the HIV prevalence ratio changes for different nonresponse HIV prevalences between males and females.

## Discussion

While biological and social factors continue to play a role in the observed difference between male and female HIV prevalence in Africa, survey nonresponders has an adverse effect on the validity of the inference one can draw from such surveys. One can make assumptions, usually unverifiable ones, in order to use statistical models to impute the information. When those assumptions are questionable, it is important to consider studying the sensitivity of inference to various models for nonresponse that do not adopt the Missing at Random assumption. In the studies that report the gender difference in HIV prevalence [11, 30, 42], sensitivity approaches to the MAR assumption such as pattern-mixture models [22] are not being reported perhaps because they are complex and hard to justify in practice. Alternatively, one can evaluate the impact the missing information has on the inference. We have chosen the latter, namely to evaluate the impact the missing information has on 29 DHSs, focusing on the sex-ratio of HIV infected individuals. Our findings strongly suggest that the data in these surveys should not be the basis for the common belief that the HIV pandemic in Africa disproportionately affects females. It is quite reasonable to conclude that there is so much nonresponse, that existing data is plausibly generated by the situation where the virus is equally distributed between the sexes.

In general, multiple imputation methods stress the importance of studying the sensitivity of inferences to various models for nonresponse [37]. Many of the studies looking at the difference in HIV prevalence between males and females fail to perform sensitivity analyses looking at multiple imputations assuming Missing Not at Random (MNAR). In practice, many researchers find the methods to perform sensitivity analyses using MNAR multiple imputation (e.g. pattern-mixture modeling) to be complex. We have provided an additional approach that can be implemented by researchers of any level.

The plausible range we present gives an indication of how the point estimate of the prevalence ratio changes when assigning subjects with an unobservable HIV outcome to be all positive or all negative. This exercise indicates how much information there is in the data, and how robust our conclusions are to the data that are missing. Overall, the point estimate of the HIV prevalence ratio is skewed to the end of the plausible interval that assigns all subjects to be HIV positive, suggesting that the HIV prevalence ratio has more flexibility to decrease towards the null than to increase away from the null when accounting for nonresponders’ HIV status. This interval can also be used to evaluate the possibility of the point estimate of HIV ratio to be close to or equal to 1.0. About three-quarters of DHS surveys had a plausible range that crosses 1.0, suggesting a plausible equivalence between male and female HIV prevalences for most countries. Even among surveys where both males and females had a high response rate (> 88%), we observed that half of those surveys had a plausible range that crossed 1.0. Furthermore, if one were to incorporate the information that these numbers result from surveys that are subject to sampling variability, wider intervals would result. While not all countries show evidence that female and male HIV prevalence is equivalent, this exercise shows the variability of this possibility across surveys. Lastly, the construction of the plausible range intervals produce intervals that are wider than one would encounter given these large-scale studies but tighter bounds would require a fabrication of questionable and tenuous assumptions. Further, if we include sampling variability in calculating any sort of bounds, such as confidence intervals, for example, we would end up with even wider bounds.

Another statistical measure that we present to address nonresponse is the prevalence ratio inflation factor. This allows an estimate of the HIV prevalence ratio that adjusts for differences in HIV prevalence between male and female nonresponders to be calculated. Unfortunately, we do not have information on HIV status of nonresponders, so it is difficult to know how this group behaves, but this ratio can be studied to see the potential for change. There have been some studies that show that nonresponders behave differently from responders [24]. It might be interesting to identify factors that contrast the nonresponders from the responders [12] and methods to estimate the HIV prevalence of nonresponders [20] to complete the story. Additional limitations which could be addressed by future work include: incorporating the reason for refusal to provide a blood sample for HIV testing and extending these methods to evaluate the impact of differential nonresponse on the standard error of the point estimate.

Future studies could expand on the plausible range interval by considering different endpoints that are not all negative test results and not all positive HIV test results. For example, one can consider a scenario where 75% of males and females had an HIV positive test result from which new plausible range intervals could be constructed. Placing a distribution(s) on this unknown parameter would yield credible intervals for the parameters of interest. Also, our proposed statistics only evaluated nonresponse for subjects that agreed to interview but did not agree to HIV testing. The three proposed statistics can be applied to the scenario where subjects do not agree to interview at all. Lastly, future studies should evaluate the robustness of the three measures using simulated data.

## Conclusions

Methods described in this paper evaluate the *reported* sex difference in HIV prevalence from 29 DHSs, without the probably unwarranted assumption of “data missing at random” to create data not gathered. Our analyses demonstrate the large impact that existing differential HIV testing nonresponse between males and females can play on HIV prevalences and especially on sex driven prevalence ratios in Africa. Indeed, it is of such magnitude that one can make a plausibly, qualitatively different conclusion from the data than has been made in the past, when the missing data was ignored, or equally as misleading, modeled using untenable assumptions.

## Appendix

We define the plausible range to be $PR = (PR^{ - } ,PR^{ + } )$, where $PR^{ - }$ is the prevalence ratio between females and males when all missing observations are assigned a negative HIV status. If we define the prevalence of the population when all female nonresponders have a negative HIV outcome as $p_{ - }^{F}$, then we can show

$$ p_{ - }^{F} = p_{r}^{F} (1 - w^{F} ) $$

because $p_{nr}^{F}$ is zero. A similar derivation can be produced for $p_{ - }^{M}$ (the HIV prevalence when all male nonresponders are designated a negative HIV test result). The $PR^{ - }$ then becomes

$$ \begin{aligned} PR^{ - } & = \frac{{p_{ - }^{F} }}{{p_{ - }^{M} }} = \frac{{p_{r}^{F} (1 - w^{F} )}}{{p_{r}^{M} (1 - w^{M} )}} \\ {\text{PR}}^{ - } & = {\text{RR}}_{\text{obs}} \times \frac{{(1 - {\text{w}}^{\text{F}} )}}{{(1 - {\text{w}}^{\text{M}} )}}, \\ \end{aligned} $$

where $RR_{obs}$ is the observed sex risk ratio if we ignore the missing data. Similarly for $PR^{ + }$, the risk ratio between females and males when all missing observations are set to positive HIV status is

$$ PR^{ + } = \frac{{p_{r}^{F} (1 - w^{F} ) + w^{F} }}{{p_{r}^{M} (1 - w^{M} ) + w^{M} }}. $$

Now we derive the prevalence ratio inflation factor. We show that true HIV prevalence ratio between females and males ($RR_{adj}$) can be expressed as

$$ RR_{adj} = RR_{obs} \times \left[ {\frac{{1 + w^{F} (R^{F} - 1)}}{{1 + w^{M} (R^{M} - 1)}}} \right]. $$

From (1) we know that the female HIV prevalence can be written as

$$ p^{F} = p_{nr}^{F} w^{F} + p_{r}^{F} (1 - w^{F} ) $$

Dividing by $p_{r}^{F}$ on both sides yields

$$ \frac{{p^{F} }}{{p_{r}^{F} }} = \frac{{p_{nr}^{F} }}{{p_{r}^{F} }}w^{F} + (1 - w^{F} ) $$

Defining $R^{F} = p_{nr}^{F} /p_{r}^{F}$ and rearranging terms, we get

$$ \frac{{{\text p}^{\rm F} }}{{{\text p}_{\rm r}^{\rm F} }} = 1 + {\text w}^{\rm F} ({\text R}^{\rm F} - 1), $$

which is equivalent to,

$$ p^{F} = p_{r}^{F} \left[ {1 + w^{F} (R^{F} - 1)} \right]. $$

The same calculations yield,

$$ p^{M} = p_{r}^{M} \left[ {1 + w^{M} (R^{M} - 1)} \right] $$

If we define $RR_{adj}$ to be the population HIV prevalence ratio comparing females to males, we get

$$ RR_{adj} = \frac{{p^{F} }}{{p^{M} }} = \frac{{p_{r}^{F} \left[ {1 + w^{F} (R^{F} - 1)} \right]}}{{p_{r}^{M} \left[ {1 + w^{M} (R^{M} - 1)} \right]}} $$

which is equivalent to our claim

$$ {\text{RR}}_{\text{adj}} = {\text{RR}}_{\text{obs}} \times \left[ {\frac{{1 + {\text{w}}^{\text{F}} ({\text{R}}^{\text{F}} - 1)}}{{1 + {\text{w}}^{\text{M}} ({\text{R}}^{\text{M}} - 1)}}} \right], $$

where $RR_{obs} = p_{r}^{F} /p_{r}^{M}$, the HIV prevalence ratio among responders.

## Abbreviations

DHS: demographic and health surveys
HIV: human immunodeficiency virus
MAR: missing at random

## References

[1] Berkley S, Naamara W, Okware S, Downing R, Konde-Lule J, Wawer M, Musagaara M, Musgrave S (1990). AIDS and HIV infection in Uganda-are more women infected than men?. Aids.

[2] Boerma JT, Ghys PD, Walker N (2003). Estimates of HIV-1 prevalence from national population-based surveys as a new gold standard. Lancet.

[3] Bouvet E, De Vincenzi I, Ancelle R, Vachon F (1989). Defloration as risk factor for heterosexual HIV transmission. The Lancet.

[4] Brick JM, Kalton G (1996). Handling missing data in survey research. Stat Methods Med Res.

[5] Brookmeyer R (2010). Measuring the HIV/AIDS epidemic: approaches and challenges. Epidemiol Rev.

[6] Clark SJ, Houle B (2014). Validation, replication, and sensitivity testing of Heckman-type selection models to adjust estimates of HIV prevalence. PLoS ONE.

[7] Cochran WG (1977). Sampling techniques.

[8] Cochran WG, Mosteller F, Tukey JW (1953). Statistical problems of the Kinsey report. J Am Stat Assoc.

[9] Corsi DJ, Neuman M, Finlay JE, Subramanian SV (2012). Demographic and health surveys: a profile. Int J Epidemiol.

[10] Fideli ÜS, Allen SA, Musonda R, Trask S, Hahn BH, Weiss H, Mulenga J, Kasolo F, Vermund SH, Aldrovandi GM (2001). Virologic and immunologic determinants of heterosexual transmission of human immunodeficiency virus type 1 in Africa. AIDS Res Hum Retrovir.

[11] Garcia-Calleja JM, Gouws E, Ghys PD (2006). National population based HIV prevalence surveys in sub-Saharan Africa: results and implications for HIV and AIDS estimates. Sex Transm Infect.

[12] Giordano K, Bärnighausen T, McGrath N, Snow R, Harlow S, Newell ML (2012). Factors associated with repeated refusal to participate in longitudinal population-based HIV surveillance in rural South Africa: an observational study, regression analyses. J HIV AIDS Surveill Epidemiol.

[13] Glynn JR, Buvé A, Caraël M, Musonda RM, Kahindo M, Macauley I, Tembo F, Study Group on Heterogeneity of HIV Epidemics in African Cities (2001). Factors influencing the difference in HIV prevalence between antenatal clinic and general population in sub-Saharan Africa. Aids.

[14] Grassly NC, Morgan M, Walker N, Garnett G, Stanecki KA, Stover J, Brown T, Ghys PD (2004). Uncertainty in estimates of HIV/AIDS: the estimation and application of plausibility bounds. Sex Transm Infect.

[15] Gregson S, Garnett GP (2000). Contrasting gender differentials in HIV-1 prevalence and associated mortality increase in eastern and southern Africa: artefact of data or natural course of epidemics?. Aids.

[16] Gregson S, Mason PR, Garnett GP, Zhuwau T, Nyamukapa CA, Anderson RM, Chandiwana SK (2001). A rural HIV epidemic in Zimbabwe? Findings from a population-based survey. Int J STD AIDS.

[17] Gregson S, Nyamukapa CA, Garnett GP, Mason PR, Zhuwau T, Caraël M, Chandiwana SK, Anderson RM (2002). Sexual mixing patterns and sex-differentials in teenage exposure to HIV infection in rural Zimbabwe. The Lancet.

[18] Hedt BL, Pagano M (2011). Health indicators: eliminating bias from convenience sampling estimators. Stat Med.

[19] Hogan DR, Salomon JA, Canning D, Hammitt JK, Zaslavsky AM, Bärnighausen T (2012). National HIV prevalence estimates for sub-Saharan Africa: controlling selection bias with Heckman-type selection models. Sex Transm Infect.

[20] Hund L, Pagano M (2013). Estimating HIV prevalence from surveys with low individual consent rates: annealing individual and pooled samples. Emerg Themes Epidemiol.

[21] Joesoef MR, Cheluget B, Marum LH, Wandera C, Ryan CA, DeCock KM, Chebet K (2003). Differential of HIV prevalence in women and men who attended sexually transmitted disease clinics at HIV sentinel surveillance sites in Kenya, 1990–2001. Int J STD AIDS.

[22] Little RJA (1993). Pattern-mixture models for multivariate incomplete data. J Am Stat Assoc.

[23] Little RJA, Rubin DB (2002). Statistical analysis with missing data.

[24] Lydié N, Robinson NJ, Ferry B, Akam E, De Loenzien M, Abega S, Study Group on Heterogeneity of HIV Epidemics in African Cities (2004). Mobility, sexual behavior, and HIV infection in an urban population in Cameroon. J Acquir Immune Defic Syndr.

[25] Magadi MA (2011). Understanding the gender disparity in HIV infection across countries in sub-Saharan Africa: evidence from the demographic and health surveys. Sociol Health Illn.

[26] Manda S, Masenyetse L, Cai B, Meyer R (2015). Mapping HIV prevalence using population and antenatal sentinel-based HIV surveys: a multi-stage approach. Popul Health Metr.

[27] Marston M, Harriss K, Slaymaker E (2008). Non-response bias in estimates of HIV prevalence due to the mobility of absentees in national population-based surveys: a study of nine national surveys. Sex Transm Infect.

[28] Mastro TD, De Vicenzi I (1996). Probabilities of sexual HIV-1 transmission. AIDS.

[29] Mishra V, Vaessen M, Boerma J, Arnold F, Way A, Barrere B, Cross A, Hong R, Sangha J (2006). HIV testing in national population-based surveys: experience from the Demographic and Health Surveys. Bull World Health Organ.

[30] Mishra V, Barrere B, Hong R, Khan S (2008). Evaluation of bias in HIV seroprevalence estimates from national household surveys. Sex Transm Infect.

[31] Moss GB, Clemetson D, D’Costa L, Plummer FA, Ndinya-Achola JO, Reilly M, Holmes KK, Piot P, Maitha GM, Hillier SL, Kiviat NC, Cameron CW, Wamola IA, Kreiss JK (1991). Association of cervical ectopy with heterosexual transmission of human immunodeficiency virus: results of a study of couples in Nairobi, Kenya. J Infect Dis.

[32] Nicolosi A, Leite MLC, Musicco M, Arid C, Gavazzeni G, Lazzarin A (1994). The efficiency of male-to female and female-to-male sexual transmission of the human immunodeficiency virus: a study of 730 stable couples. Epidemiology.

[33] Nyirenda M, Zaba B, Barnighausen T, Hosegood V, Newell M (2010). Adjusting HIV prevalence for survey non-response using mortality rates: an application of the method using surveillance data from rural South Africa. PLoS ONE.

[34] Ramirez-Avila L, Nixon K, Noubary F, Giddy J, Losina E, Walensky RP, Bassett IV (2012). Routing HIV testing in adolescents and young adults presenting to an outpatient clinic in Durban, South Africa. PLoS ONE.

[35] Redondo-Sendino Á, Guallar-Castillón P, Banegas JR, Rodríguez-Artalejo F (2006). Gender differences in the utilization of health-care services among the older adult population of Spain. BMC Public Health.

[36] Royce RA, Sena A, Cates W, Cohen MS (1997). Sexual transmission of HIV. N Engl J Med.

[37] Rubin DB (1987). Multiple imputation for nonresponse in surveys.

[38] Rutstein SO, Rojas G (2006). Guide to DHS statistics.

[39] Sindelar JL (1982). Differential use of medical care by sex. J Polit Econ.

[40] UNAIDS: Joint United Nations Programme on HIV/AIDS 2013. UNAIDS report on the global AIDS epidemic. http://www.unaids.org/sites/default/files/en/media/unaids/contentassets/documents/epidemiology/2013/gr2013/UNAIDS_Global_Report_2013_en.pdf. Retrieved 17 Feb 2015.

[41] Vanable PA, Carey MP, Blair DC, Littlewood RA (2006). Impact of HIV-related stigma on health behaviors and psychological adjustment among HIV-positive men and women. AIDS Behav.

[42] Ziraba AK, Madise NJ, Matilu M, Zulu E, Kebaso J, Khamadi S, Okoth V, Ezeh AC (2010). The effect of participant nonresponse on HIV prevalence estimates in a population-based survey in two informal settlements in Nairobi city. Popul Health Metr.

